# Applying an Equity Lens to Evidence-Based Preventive Interventions: a Systematic Review of Subgroup Findings from Experimental Evaluations

**DOI:** 10.1007/s11121-025-01765-3

**Published:** 2025-01-17

**Authors:** Pamela R. Buckley, Charleen J. Gust, Sarah Gonzalez Coffin, Sheba M. Aikawa, Christine M. Steeger, Fred C. Pampel

**Affiliations:** Institute of Behavioral Science, University of Colorado Boulder, Boulder, CO 483 UCB80309 USA

**Keywords:** Evidence-based, Health disparities, Equity, Preventive interventions, Minoritized youth

## Abstract

Evidence reveals that minoritized groups face disparities, underscoring the need for interventions to address behavioral health inequities. This review examined which minoritized populations are represented in evidence-based preventive interventions (EBPIs) and whether they equitably benefit from these programs. Using the Blueprints for Healthy Youth Development online clearinghouse, we synthesized findings from 240 high-quality experimental evaluations of EBPIs conducted in the U.S. between 2010 and 2023 and performed a descriptive analysis based on consensus coding to assess (1) the prevalence of culturally tailored EBPIs; (2) how frequently tests for subgroup effects were conducted; and (3) whether subgroup tests indicated differential benefits for minoritized groups. We found few culturally tailored interventions (31%), with 4% evaluating EBPIs developed for African American or Black populations and 1% for Hispanic or Latino youth. Additionally, only 25% and 15% tested for subgroup effects by race and ethnicity, respectively. For other subgroups, few (28%) evaluations included effects by economic disadvantage while 47% examined outcomes by binary gender categories. Essentially no reports tested for subgroup effects by sexual identity, location, or nativity status. Encouraging findings were that EBPIs more often benefited racial and ethnic minoritized groups, and there was an upward trend in reporting subgroup tests across time. EBPIs should test for subgroup effects to answer the questions of “what works for whom?” and “in which settings?” and to better understand the generalizability of findings. Investments are needed in culturally grounded programs developed for historically marginalized populations and trials of EBPIs that investigate mitigating health disparities.

**Supplementary Information:**

The online version contains supplementary material available at 10.1007/s11121-025-01765-3.

The number of people who are culturally and linguistically diverse is growing globally. In the United States (U.S.), Asian or Asian American, Black or African American, Native American or American Indian or Alaskan Native, Native Hawaiian or Pacific Islander, and Hispanic or Latino individuals (i.e., currently classified as racial and ethnic minoritized populations) are particularly susceptible to adverse physical, mental, and behavioral health outcomes (Alegria et al., 2015). This is largely due to their social determinants of health (or risk and protective factors), defined as the circumstances in which people are born, grow, work, live, and age that collectively influence well-being (WHO Commission on Social Determinants of Health & World Health Organization, 2008). Examples include access to quality education, employment opportunities, affordable housing, safe neighborhoods, clean drinking water, and nutritious food. Social determinants of health are shared across populations; as such, racial and ethnic minoritized groups carry the unique burden of structural and systemic racism that denies equitable opportunities for creating the safety and stability needed to avoid adverse experiences. Addressing these inequities is extremely complex and involves multiple strategies, such as (1) scaling equity-focused preventive interventions shown to promote healthy development and (2) structural interventions aimed at changing societal structures and policies that produce and perpetuate inequality (Boyd et al., 2023; Murry et al., 2024). Our focus is on the former, with implications for the latter.

Evidence-based interventions (EBIs) refer to specific programs, practices, or strategies that are grounded in empirical evidence derived from high-quality research studies, such as randomized controlled trials (RCTs), which provide empirical support for their efficacy, effectiveness, and safety (Gottfredson et al., 2015; Steeger et al., 2021). Evidence-based preventive interventions (EBPIs) share these attributes. However, the term “preventive” underscores their proactive nature; EBPIs intervene *before* problems occur or escalate to reduce the burden of disease and dysfunction on individuals, families, communities, and societies (Boyd et al., 2023; Fishbein & Sloboda, 2023). Strayhorn et al. (2024) define equitability of an intervention as the extent to which the health benefits provided by an intervention are distributed evenly, such that all participants have an opportunity to achieve the desired outcome of the intervention. Building upon research calling for clear reporting and better representation of racial and ethnic minoritized populations to improve the utility of preventive interventions (Buckley et al., 2023), we explore the prevalence of EBPIs that benefited minoritized youth.

The motivation behind our research is twofold. First, while EBPIs strengthen decision-making around adopting interventions, questions remain about whether they promote a good fit between services and local context. Evidence is more likely to influence decision-making when studies are implemented in familiar contexts (Mills et al., 2020). The field, however, lacks guidance in understanding the reliability of effects across diverse groups and settings (Bryan et al., 2021; Buckley et al., 2023). Insufficient attention to heterogeneity can lead to inaccurate conclusions about the effectiveness of EBPIs in different contexts (Bryon et al., 2021) and diminish scrutiny of whether interventions need cultural tailoring (Bo et al., 2023; Jackson, 2009). Overlooking heterogeneity also limits our understanding of when and how EBPIs improve the lives of those most affected by health disparities, or preventable differences in health outcomes and opportunities to achieve optimal health between groups of people (Brown et al., 2018). External validity is thus of great importance in scaling and sustaining EBPIs.

Second, many public authorities and private funders encourage states to take a more evidence-based approach to supporting behavioral health services. Referred to as “evidence-based decision-making,” this method uses empirical evidence in a systematic way to guide funding decisions (Schindler, 2024). This study addressed an issue of fundamental importance — whether there is a sufficient evidence base of EBPIs that benefit minoritized populations. Such evidence can inform accountability, facilitate the scale-up of successful interventions, and promote health equity to achieve population-level impacts.

## Culturally Tailored Interventions

Concerns about the widespread dissemination of interventions that are tested for one sociodemographic group but uncritically applied to others (Murry et al., 2024; Pina et al., 2019; Thier et al., 2020) stem from the historical tendency in the U.S. to develop and/or implement EBPIs with a current majority population in mind (White of Western European descent; Buckley et al., 2023). This has led to oversight of the profound influence that culture, race, ethnicity, and English language proficiency have on outcomes. Culturally tailored interventions, designed with consideration given to the strengths and experiences of the target population to enhance program relevance, engagement, and effectiveness, seek to address this omission. These interventions occur along a continuum ranging from (1) culturally adapted interventions, to (2) deep structure adapted interventions, to (3) culturally grounded interventions (Okamoto et al., 2014).

Culturally adapted interventions involve modifications to fit cultural, linguistic, and/or contextual needs while maintaining core components (Barrera et al., 2017; Torres-Ruiz et al., 2018). For instance, *Functional Family Therapy* (*FFT*) is a selective EBPI helping youth with mild to severe behavior problems overcome delinquency, substance abuse, and violence. In 2009, researchers adapted FFT for gang-involved youth as an indicated prevention program to reduce crime, given the large body of research establishing inverse relationships between parental supervision and monitoring of youth activities and gang membership and the strong correlation between gang membership and criminal offending (Pyrooz et al., 2016). FFT core components remained unchanged; rather, accommodations were made to the frequency (e.g., 20 sessions over 4 months versus FFT’s 12 sessions over 3 months) and duration of sessions to better address risk factors typically more salient among gang than delinquent populations (see Table 1 of Pyrooz & Buckley, 2023). A high-quality RCT (as defined by Steeger et al., 2021) in a sample of 129 families with adjudicated males (15% gang-involved) revealed reductions for intervention youth (compared to control) in recidivism over 18 months (Gottfredson et al., 2018).

**Table 1:** *N*s and proportions of reports evaluating culturally tailored EBPIs by subgroup

	Sample		
	Full^b^	Analysis^c^	Sub-anal ysis^d^
	(*N* = 292)	(*N* = 240)	(*N* = 100)
	*n* (Prop)	*n* (Prop)	*n* (Prop)
Culturally tailored EBPI^a^	82 (.28)	75 (.31)	25 (.25)
Target population of culturally tailored EBPI^a^			
Race			
Asian or Asian American Black or African American Native American, American Indian, Alaska Native Native Hawaiian or Pacific Islander White	0 (.00) 9 (.03) 0 (.00) 0 (.00) 0 (.00)	0 (.00) 9 (.04) 0 (.00) 0 (.00) 0 (.00)	0 (.00) 1 (.01) 0 (.00) 0 (.00) 0 (.00)
Ethnicity — Hispanic or Latino	2 (.01)	2 (.01)	0 (.00)
Gender (dichotomy of male or female)	43 (.15)	36 (.15)	3 (.03)
Economic disadvantage	25 (.09)	25 (.10)	17 (.17)
Location — urban	8 (.03)	8 (.03)	4 (.04)
Location — rural	6 (.02)	6 (.02)	1 (.01)

*EBPIs*, evidence-based preventive interventions

^a^EBPIs may target multiple groups so that the proportions for the targeted groups sum to more than the total of culturally tailored EBPIs in the first row

^b^Reports conducted both within and outside of the United States

^c^Reports conducted in the United States

^d^Reports conducted in the United States that tested for one or more of the following subgroups: race, ethnicity, gender, sexual identity, economic disadvantage, location (rural, urban, suburban), nativity status (foreign-born — yes/no)

Recognizing that cultural differences may require more fundamental adjustments to achieve meaningful impact, deep structure adapted interventions modify the underlying theoretical framework of an intervention to better align with the cultural context of the target population (Bo et al., 2023). For example, *Nuestras Familias: Andando Entre Culturas* is a parent training program for Spanish-speaking Hispanic or Latino families residing in a U.S. emerging immigration context designed to help adolescents respond effectively to the risks of substance use and unsafe sexual behavior (Martinez et al., 2022). The program was developed through a collaborative effort between a social service agency serving Latino clients, community partners, and the Oregon Social Learning Center (OSLC) to incorporate empirically supported core components from the OSLC’s GenerationPMTO parent-training EBPI (Forgatch & DeGarmo, 1999) with elements that address culturally relevant experiences of Latino families (acculturation stress, discrimination, etc.). Focus groups were conducted with Latino parents to examine the program’s cultural validity (Domenech Rodriguez et al., 2011; Martinez & Eddy, 2005). A high-quality RCT (see Steeger et al., 2021) with 241 Spanish-speaking families found that at posttest, compared to the control group, intervention youth reported significantly lower likelihoods of tobacco and illicit drug use and had fewer parent-reported internalizing behaviors (Martinez et al., 2022).

Culturally grounded programs are developed with a specific culture in mind to reflect the target population’s values, behaviors, and norms. These programs incorporate cultural considerations into their design, ensuring they are intrinsically aligned with the lived experiences and cultural contexts of the populations they serve (Bo et al., 2023). For instance, the *Arrowhead Business Group* is a strengths-based entrepreneurship education program for adolescents from under-resourced communities. Co-created through a tribal-university partnership between the White Mountain Apache Tribe in northeastern Arizona and the Johns Hopkins Center for American Indian Health, the program fosters connectedness to caring for adults, healthy peers, and school and aims to improve behavioral health and foster economic development (Tingey et al., 2016). A high-quality RCT (see Steeger et al., 2021) preregistered at ClinicalTrials.gov (#NCT02157493) that included 394 Native American youths residing at the Fort Apache Reservation found at posttest, compared to control, intervention youth showed lower marijuana use, and fewer school days missed and fights on school property (Tingey et al., 2020a, b).

## Theoretical Framework

This research focused on differential outcomes by distinct socially constructed subgroups of race and ethnicity, since racial and ethnic minoritized groups carry the unique burden of structural and systemic racism. We also examined individual subgroups by gender and economic disadvantage, since they are commonly reported in preventive intervention research (Tinner et al., 2023), and of nativity status (i.e., native- versus foreign-born) and sexual identity when available. Our work was framed by the concept of equity — the state or ideal of being just and fair — which is a structural concept achieved when *all* individuals have access to the essentials needed for success. This view stresses the need to address individual differences at the outset (proximal level) and entrenched systemic barriers (distal level) to ensure equal opportunities (Boyd et al., 2023; Murry et al., 2024).

## How the Study Complements and Advances Empirical Research

Previous research demonstrates the need to assess racial and ethnic heterogeneity in intervention development, evaluation, and dissemination (Murry et al., 2024). For example, Gaias et al. (2020) revealed that 27% of educational intervention studies meeting What Works Clearinghouse (WWC) standards failed to report participants’ race or ethnicity. Buckley et al. (2023) identified similar research gaps in the Blueprints for Healthy Youth Development (i.e., Blueprints) online clearinghouse database, emphasizing the need for improved reporting of sample characteristics in youth preventive intervention research to advance EBPIs that reduce racial disparities and determine whether communities with unique demographic features (e.g., rural location, specific racial and ethnic groups) have been studied.

Further, in a systematic review and meta-analysis of 30 RCTs, Bo et al. (2023) found that culturally tailored EBPIs were effective in reducing substance use among U.S. Black, Hispanic, and Native American adolescents. The authors, however, called for more high-quality research to replicate these findings and advance the development of EBPIs for multiracial, Asian American, and Pacific Islander adolescents. Meanwhile, interventions that are not culturally tailored (i.e., 78% of preventive interventions in Buckley et al., 2023) may work better for certain groups. For instance, a high-quality RCT (Steeger et al., 2021) of Accelerated Study in Associate Programs (ASAP), which addresses barriers for college students from low-income backgrounds, indicated higher rates of degree completion for the ASAP group compared to the control group, with larger effects for Black and White students than Hispanic or Latino students (Weiss et al., 2019). While subgroup tests are typically exploratory, they provide valuable insight into group differences and are therefore important to study (Bloom & Michalopoulos, 2013).

Amuta-Jimenez et al. (2020) offered a potential solution for examining how the design, testing, and dissemination of EBPIs may perpetuate injustice, as well as how EBPIs can be radically re-envisioned to promote health equity for historically marginalized communities and populations: synthesize findings from experimental evaluations to assess heterogeneity and whether EBPIs may differentially affect minoritized populations (Murry et al., 2024). *That is our aim*. Building on previous research (Buckley et al., 2023; Gaias et al., 2020), we explore the differential impacts of EBPIs on youth behavioral health outcomes.

## Subgroup Analysis: Testing How Effects Vary

Subgroup analysis compares outcomes for subsets of participants defined by certain characteristics such as baseline risk factors (e.g., hyperactivity), time periods (i.e., cohort effects), and demographic characteristics like race, ethnicity, SES, and location (e.g., rural, urban; Bloom & Michalopoulos, 2013). Though useful for determining the extent to which positive findings are broadly distributed across subgroups (suggesting robust effects), the use of subgroup analysis for another goal — to investigate an intervention’s potential to reduce or exacerbate health disparities — is gaining momentum (Gaias et al., 2020; Tinner et al., 2023). Often referred to as tests for interactive or moderator effects, these analyses assess whether intervention impacts vary *between* group characteristics by including interaction terms (e.g., race x treatment condition) as part of the omnibus testing procedure. Alternatively, separate models by subgroup, which involve disaggregation of intervention results by sociodemographic characteristic, establish whether the program is effective *within* each group for which a model is estimated. This distinction is important because reducing health disparities requires more than showing within-group effects. Unless EBPIs serve homogeneous samples wherein (for example) 75% or more of the sample is from a minoritized group (Huey & Polo, 2008), health equity requires significant between-group effects favoring minoritized groups (Boyd et al., 2023).

The appropriateness of subgroup analysis depends on the rationale and defining characteristics that are, at a minimum, prespecified (Fayers & King, 2009). Ideally, subgroup analyses should be included in the preregistration process, thereby establishing a time-stamped record of the researcher’s intentions and ensuring transparency and accessibility regarding the study’s design and procedures. Along with other advantages, preregistration discourages cherry-picking of results (i.e., *p*-hacking) and in principle should clarify whether the study is adequately powered to detect subgroup differences (Bloom & Michalopoulos, 2013; Elliott et al., 2022).

## Current Study

Using data from Blueprints, an online clearinghouse of EBPIs for youth (Mihalic & Elliott, 2015), we conducted a systematic review (Munn et al., 2018) and descriptive analysis of report-level data from experimental evaluations published between 2010 and 2023 that met Blueprints evidence standards for a high-quality design (see Table 1 of Steeger et al., 2021). Our goal was to identify the evidence base of EBPIs that benefit minoritized populations. The decision to rely solely on Blueprints was motivated by budget limitations and our unlimited access to the Blueprints database. Our primary research questions were: (1) How prevalent are culturally tailored EBPIs? (2) How often do evaluation studies test for differences in program effects by race, ethnicity, gender, sexual identity, economic disadvantage, location, and/or nativity status, and do these frequencies differ for culturally tailored EBPIs? (3) How prevalent are evaluation studies showing beneficial effects for minoritized groups, and do these frequencies differ for culturally tailored EBPIs? Our secondary research questions were: (4) Has the proportion of studies that examine differences in effects by these groups increased over time, and does the change differ for culturally tailored EBPIs? (5) For studies examining differences in effects by these groups, how frequently are the subgroup analyses preregistered, and do the frequencies differ for culturally tailored EBPIs?

## Methods

### Eligibility

This review followed Blueprints’ definition of a program, study, and report. Blueprints defines a program as an intervention with a prespecified set of activities that have the goal of improving youth behavioral outcomes. Blueprints defines a study as an empirical evaluation of a program using a distinct design and sample. Programs in the Blueprints database can have multiple studies, as investigators use different samples and methods to evaluate a program. A study may have one or more reports. Multiple reports within a study examine the same program and use the same sample but cover different outcomes, follow-up periods, and/or mediator or moderator analyses. Blueprints identifies sample characteristics within a study and analysis characteristics within a report.

Our eligibility criteria, therefore, included reports that (1) were part of the Blueprints database (see Online Resource Panel 1), (2) were published between January 2010^1^ and September 2023, and (3) were identified as having implemented a high-quality experimental design (i.e., met Blueprints standards of evaluation quality as outlined in Steeger et al., 2021) with findings that demonstrate consistent and statistically significant positive effects and no evidence of harmful main effects. These reports are certified by Blueprints as “what works,” i.e., an EBPI (Mihalic & Elliott, 2015; Steeger et al., 2021). Non-certified reports (did not meet standards for “what works”) with strong internal validity were included if they found no or very few effects on behavioral outcomes or used a very small or specialized sample (e.g., pilot group design studies that are often underpowered to detect differences). In short, eligible reports had high-quality methodology and appropriate analyses regardless of results.

### Search Strategy

Our search strategy followed the same systematic procedure that Blueprints employed from 2010 to 2023 as part of its standard operating procedures. Reports were screened into our sample through the three-step Blueprints review process involving (1) identification using a systematic search (see Online Resource Panel 2 for a description of Blueprints’ search strategy, and Online Resource Panel 3 for Blueprints’ search term clauses), (2) a risk of bias assessment (see Table 1 of Steeger et al., 2021) conducted by Blueprints staff (internal review), and (3) certification determined by Blueprints scientific advisory board (external review). Steeger et al. (2021) detailed Blueprints’ internal and external review process.

### Sample

**Fig. 1:**
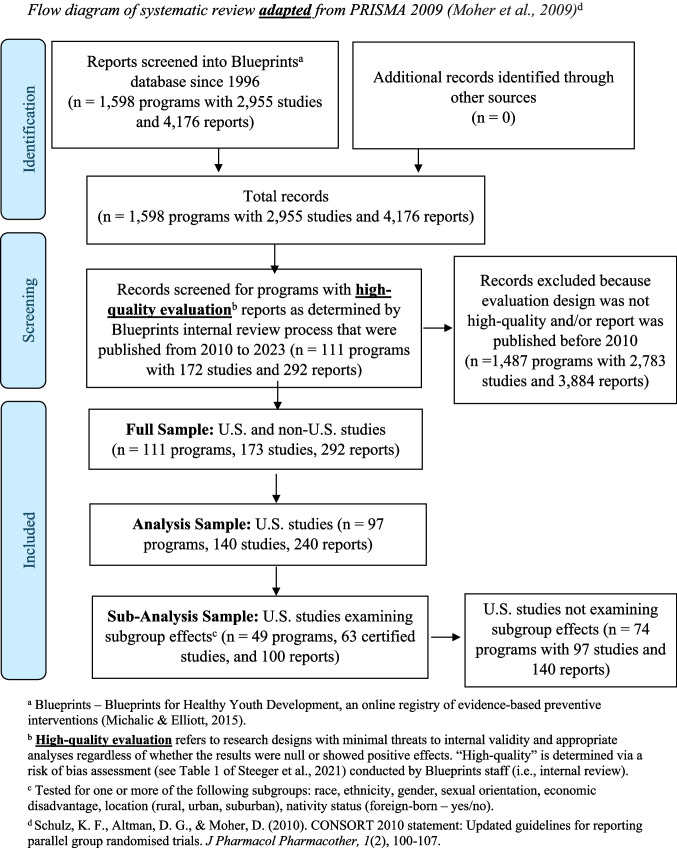
Flow diagram of systematic review **adapted** from PRISMA 2009 (Moher et al., 2009)^d^. ^a^Blueprints — Blueprints for Healthy Youth Development, an online registry of evidence-based preventive interventions (Michalic & Elliott, 2015). ^b^**High-quality evaluation** refers to research designs with minimal threats to internal validity and appropriate analyses regardless of whether the results were null or showed positive effects. “High-quality” is determined via a risk of bias assessment (see Table 1 of Steeger et al., 2021) conducted by Blueprints staff (i.e., internal review). ^c^Tested for one or more of the following subgroups: race, ethnicity, gender, sexual orientation, economic disadvantage, location (rural, urban, suburban), nativity status (foreign-born — yes/no). ^d^Schulz, K. F., Altman, D. G., & Moher, D. (2010). CONSORT 2010 statement: Updated guidelines for reporting parallel group randomised trials. *J Pharmacol Pharmacother, 1*(2), 100–107

Our sample was selected as follows (Fig. 1). As of September 2023, when a list of existing reports was created for coding and analysis, the Blueprints database contained 1,598 programs added since the clearinghouse’s inception in 1996. Nested within the 1,598 programs were 2,955 studies, and nested within the 2,955 studies were 4,176 reports. Selecting programs that met the eligibility criteria for this review resulted in a full sample of 111 programs with 172 studies and 292 reports conducted within and outside of the U.S. Our analysis sample focused on 97 programs with 140 studies and 240 reports conducted in the U.S., since the reports done outside the U.S. (*n* = 52, 18%) occurred in contexts where the socially assigned meanings of race, ethnicity, and other characteristics differ. From the analysis sample, we also created a sub-analysis sample of 49 programs, 63 studies, and 100 reports that tested for subgroup effects.

### Coding

Development of the codebook proceeded in three stages. First, the study used the instrument from Buckley et al. (2023) to code for (1) sample-level characteristics (see Online Resource Panel 4) and (2) the evaluation of a culturally tailored intervention (with culturally adapted, deep structure adapted, and culturally grounded interventions coded as “culturally tailored”), in which populations were served based on race, ethnicity, gender or gender identity, economic disadvantage, geographic location, and/or nativity status. A program was coded “culturally tailored” only if one of these groups was part of the name and/or if the logic model, theory of change, and/or description specified it was developed for at least one of these groups.

Second, drawing from previous research (Buckley et al., 2022), two co-authors (P.B., C.G.) developed transparency codes to assess whether studies had preregistered subgroup analyses. Third, three of the study co-authors (P.B., C.G., F.P.) developed new codes to determine if and how reports tested for subgroup effects. The codes distinguished moderation or interaction tests that compared subgroup effects from between-group or within-group tests for subgroups. The former tests show subgroup effects relative to a comparison group, while the latter tests show absolute subgroup effects. The tests differ in strategy; both are recommended (Bloom & Michalopoulos, 2013) but reports often use one or the other (and sometimes both). Codes were refined via an iterative process through discussion and several pilot testing rounds.

All reports were double coded by three teams of rotating pairs to ensure consistency (McHugh, 2012). The first team (C.G., S.G.C., S.A.) screened the full sample (*n* = 292) for reports of EBPIs that examined subgroup effects (sub-analysis sample). The sub-analysis sample was coded for subgroup tests by the second team (F.P., C.G.) and transparency by the third team (P.B., S.G.C., C.S.). All reports were independently coded and entered into an online form. Pairs of codes were compared, and discrepancies were discussed until reaching consensus.

### Analysis

Analyses were conducted at the report level. We ran frequencies for all analyses but added chi-square tests for the comparisons across culturally tailored interventions and time. Given the descriptive nature of this research, we sometimes summed together counts for reports that conducted interaction (moderator) analysis and within-group analysis but more generally distinguished between the two analyses. For research question 3 on beneficial effects, interaction (moderation) tests were counted as beneficial when the interaction terms showed a statistically significant, more favorable effect for minoritized youth. For within-group tests, beneficial effects were counted as favoring minoritized youth when — for example in testing race or ethnicity — the within-group results included a statistically significant positive outcome for a non-White group (i.e., Asian or Asian American, Black or African American, Native American or American Indian or Alaskan Native, Native Hawaiian or Pacific Islander, and Hispanic or Latino individuals).

## Results

As background, Online Resource Table 1 describes the characteristics of our sample. Reports in the full (*n* = 292), analysis (*n* = 240), and sub-analysis (*n* = 100) samples included mostly RCTs (68–73%) and cluster RCTs (23–27%), with some high-quality QEDs (3–6%; e.g., propensity score matching and instrumental variable analysis). Most reports were published in an academic journal (versus the grey literature); however, 59% of reports in the sub-analysis sample were published in a journal compared to 77% for the full and 73% in the analysis sample. Thus, in our sample, reports from the grey literature conducted subgroup tests at higher rates than reports published in academic journals. Across all three samples, most reports evaluated interventions delivered in various settings targeting ages 5–24 years. Most EBPIs were delivered in schools, with 68% of reports in the sub-analysis sample conducted in a school setting compared to 60% for the full and 59% in the analysis sample. Outcomes across the three samples included educational attainment (36–59%), problem behavior (34–53%), emotional well-being/mental health (19–16%), positive relationships (2–4%), and physical health (2–3%).

In addition, Online Resource Table 2 details sample characteristics of reports in our review. For the analysis sample (*n* = 240 reports), 88% provided details on the racial composition, in which most enrollees were White (36%) followed by Black or African American (31%), and 27% collapsed across race or categorized race with ethnicity. Meanwhile, 74% reported ethnicity, with 24% of enrollees identifying as Hispanic or Latino. American Indian or Alaskan Native, Asian American, and Native Hawaiian or Pacific Islander populations were not well represented in EBPI samples or were combined in a residual “other” category. Findings were similar for the full and sub-analysis samples.

**Table 2:** Proportions of reports testing for subgroup effects in U.S. sample (*n* = 240 reports) by nine subgroups

	1	2	3	4	5	6	7	8	9
I. Tested subgroups									
No Yes	.754 .246	.679 .321	.854 .146	.795 .205	.533 .467	.992 .008	.721 .279	.983 .017	1.000 .000
II. RQ 2: How often did evaluation studies of EBPIs test for differences in program effects by race, ethnicity, gender, sexual identity, economic disadvantages, location, and nativity subgroups?*									
a. Examined interaction	.046	.100	.025	.067	.138	.008	.042	.000	.000
b. Examined within-group	.033	.042	.038	.038	.042	.000	.033	.008	.000
c. Examined both	.058	.071	.054	.071	.138	.000	.042	.008	.000
d. Homogeneous sample	.108	.108	.029	.029	.150	.000	.163	.000	.000
III. RQ 3: How prevalent were evaluation studies of EBPIs showing beneficial effects for race, ethnicity, gender, sexual identity, economic disadvantages, location (urban, rural, suburban), and nativity subgroups?*									
e. Relative benefit in interaction	.029	.079	.021	.050	.125	.008	.038	.000	.000
f. Absolute benefit in within group	.029	.038	.029	.029	.025	.000	.033	.008	.000
g. Benefit in interaction and within group	.054	.058	.033	.042	.121	.000	.038	.004	.000
h. Homogeneous sample	.108	.108	.029	.029	.150	.000	.163	.000	.000
i. No benefit in interaction or within group	.025	.038	.038	.054	.042	.000	.008	.004	.000

*Proportions in panels II and III are based on the number of reports that tested for subgroups (i.e., coded “yes” in panel I). **Homogeneous sample** — more than 75% of the sample came from the designated minoritized group (i.e., a method used in Huey & Polo, 2008). **Subgroup legend:**

^1^Measured **race** according to one or more of the U.S. Census categories of Asian or Asian American, Black or African American, Native American or American Indian or Alaska Native, Native Hawaiian or Pacific Islander, or Multiracial

^2^Measured race according to one or more of the U.S. Census categories (see #1) or to collapsed categories

^3^Measured **ethnicity** according to one or more of the U.S. Census categories of Hispanic or Latino

^4^Measured ethnicity according to the U.S. Census categories (see #3) or mixed categories with racial groups

^5^Measured **gender** according to a binary category of female

^6^Measured **sexual identity**, including lesbian, gay, bisexual, transgender, queer or questioning, intersex, asexual, and more (LGBTQIA +)

^7^Measured **economic disadvantage** categorized as youth that fell within the government’s poverty level as measured by different metrics, such as the percentage of students that qualified for free or reduced-price lunch (a proxy in education research for families in the U.S. that are low-income)

^8^Measured **location** where the study was conducted (urban, suburban or rural)

^9^Measured **nativity status** (i.e., foreign-born in the country where the study was conducted)

For research question 1, we examined the prevalence of reports evaluating culturally tailored EBPIs. Focusing on the analysis sample (*n* = 240), the findings showed that 31% (*n* = 75) evaluated EBPIs that were culturally tailored (Table 1). Of these 75 reports, 4% (*n* = 9) were developed for Black or African American youth, 1% (*n* = 2) for Hispanic or Latino populations, 15% (*n* = 36) for a specific gender (i.e., male–female dichotomy), 10% (*n* = 25) for youth experiencing economic disadvantage, and 3% (*n* = 8) for urban and 2% (*n* = 6) for rural youth. Findings were consistent across full and sub-analysis samples.

For research question 2, we examined the prevalence of subgroup analyses, focusing on the analysis sample (Table 2). Panel I of the table shows the proportions of the 240 U.S. reports that presented subgroup analyses in the form of either interaction or within-group tests. They ranged from 47% of reports that tested for subgroups by gender (i.e., male–female dichotomy) to essentially none that tested for subgroups by sexual identity or nativity status. For race, ethnicity, and economic disadvantage, the proportions ranged from 15 to 32%. In short, most reports did not attend to subgroups. For reports that tested subgroups, these effects came in different forms. Some tested for relative subgroup differences in effects using interaction terms or moderation analyses (1a–9a in Table 2), some tested for absolute subgroup effects using within-group analyses (1b–9b), some did both (1c–9c), and some used homogeneous samples, defined as when 75% or more of the sample was from the designated minoritized group (Huey & Polo, 2008; 1d–9d). Most common were reports with homogenous samples of minoritized groups, primarily racial minoritized groups (11%; see 1d), females (15%; see 5d), and economically disadvantaged groups (16%; see 7d). Reports that tested subgroups were distributed in similar proportions across the three methods, and each of the three methods was used rarely. Subgroup tests were more common for race and ethnicity when counting reports that used mixed categories such as “non-White” or “minority” (2a–2c and 4a–4c).

For research question 3, we examined the prevalence of reports that showed beneficial effects (Panel III of Table 2). Findings reflect the number of reports demonstrating differential effects as a proportion of the number of reports that performed subgroup analyses (i.e., coded “yes” in Panel I of Table 2), rather than as a proportion of all 240 studies in the analysis sample. Beneficial effects refer to a significant interaction effect favoring the subgroup, a significant within-group effect for the subgroup, or both. For these reports, the proportions showed that EBPIs more often benefited minoritized groups than not. For example, 11% of the reports found beneficial effects for racial minoritized youth using heterogeneous samples (summing 1e, 1f, and 1 g), and 11% examined homogenous samples for these groups (see 1 h), while 3% found no benefit or benefits for White youth only (see 1i). Similarly, for ethnicity, 8% found beneficial effects for Latino or Hispanic youth using heterogeneous samples (summing 3e, 3f, and 3 g), and 3% examined homogenous samples (see 3 h), while 4% found no benefit or benefits for non-Hispanic youth only (see 3i). For gender, 27% found beneficial effects for females using heterogeneous samples, 15% found beneficial effects using homogeneous samples, and 4% found no beneficial effects or effects for males. For economic disadvantage, 11% found beneficial effects for economically disadvantaged groups using heterogeneous samples, 16% found beneficial effects using homogenous samples, and 1% found no beneficial effects or effects for economically advantaged groups.

Research questions 2 and 3 also inquired about culturally tailored programs (Online Resource Table 3). The table uses three categories for each type of subgroup: (1) did not examine subgroup differences, (2) used a homogenous sample, or (3) examined subgroup differences. Although rare, the proportions show that culturally tailored programs commonly used a homogeneous sample. For example, focusing on the analysis sample, 8% of the 231 reports for non-culturally tailored programs used a racially homogenous sample, while 89% of the 9 reports for culturally tailored programs used a racially homogenous sample. Given the focus on a single group, reports evaluating culturally tailored EBPIs seldom tested subgroup differences.

For research question 4, since our time frame ranged from 2010 to 2023, we divided this 13-year period in half and reviewed the proportion of reports that examined subgroup differences: (1) earlier (2010 to 2016), or (2) later (2017 to 2023). The sample sizes were too small to perform separate analyses for culturally tailored EBPIs. Significant time differences emerged for race and ethnicity. More reports examined subgroup effects in the later than earlier period (Online Resource Table 4). Findings were similar across all three samples.

Research question 5 focused on preregistration (Online Resource Table 5). Of the 100 U.S. reports that tested subgroups, 21% were registered retrospectively, and 2% were registered prospectively. Seven percent registered their subgroup analysis, with 6% doing so retrospectively and 1% doing so prospectively. Seventy-seven percent did not register their analysis at all. We found no significant differences in registration, preregistration, or subgroup registration for reports of culturally tailored and non-culturally tailored EBPIs.

## Discussion

We reviewed the literature on 97 separate EBPIs for youth with 140 studies (each study examined one sample of youth) and 240 reports (as multiple reports can come from the same sample) conducted in the U.S. and published between 2010 and 2023. Like Gaias et al. (2020), who reviewed education intervention reports meeting What Works Clearinghouse (WWC) standards, we described the prevalence of equity-promoting EBPIs in the Blueprints for Healthy Youth Development database (regardless of whether reducing disparities was a focus of the intervention’s theoretical rationale). Replicating findings from Buckley et al. (2023), we found a low prevalence of culturally tailored interventions (31%, *n* = 75 reports), with only 4% (*n* = 9) evaluating EBPIs developed for African American or Black populations and less than 1% (*n* = 2) evaluating EBPIs for Hispanic or Latino youth.

Moreover, EBPIs with demonstrated promise for mitigating behavioral health disparities were limited. Consistent with Gaias and colleagues (2020) discovery that very few reports (i.e., 19% in their sample) included subgroup analyses assessing an intervention’s potential to reduce racial and ethnic educational disparities, we found that only 25% (*n* = 60) of the 240 U.S. reports included subgroup or interaction effects by race, measured as one or more of the following categories: Asian or Asian American, Black or African American, Native American or American Indian or Alaska Native, Native Hawaiian or Pacific Islander, or Multiracial. Meanwhile, 15% (*n* = 36) of reports included effects by ethnicity, measured as Hispanic or Latino. Also, subgroup or interaction tests were more common when counting reports that used mixed and non-specified categories such as “non-White” or “minority.” Specific racial and ethnic categories are preferred over collective terms, but if the numbers are small and require collapsing across groups, categories should be explicitly defined to inform generalizability (Flanagin et al., 2021). Regarding other subgroups of interest, 28% (*n* = 67) of reports included effects by economic disadvantage, and almost half (47%, *n* = 110) examined outcomes by gender (measured in binary categories of male and female). Essentially no reports tested for subgroup or interaction effects by sexual identity, location (urban, suburban, rural), or nativity status.

Our findings did, however, show some encouraging patterns among the few reports that tested subgroups. For example, EBPIs more often benefited racial and ethnic minoritized groups. We also observed an upward trend in reporting subgroup effects across time. However, concerns over the cherry-picking of results surfaced, as subgroup analyses were registered in only 7% (*n* = 7) of the 100 reports examining subgroups, with only 1% doing so prospectively.

### Implications

The present findings indicate a dearth of evidence regarding the potential impact of EBPIs on pervasive behavioral health disparities. In addition to recruitment and detailed reporting of diverse samples (Buckley et al., 2023), researchers should conduct preregistered analyses that specifically investigate main effects (i.e., “what works), but also preregister subgroup tests to examine for whom and under what conditions an EBPI is likely to produce results. Although reports showing no subgroup effects have historically been viewed as important for broadly improving outcomes, there is growing acceptance that no “silver bullet” exists (Schindler, 2024). Interventions that are effective overall but not equitable (e.g., benefits are concentrated among those who are already advantaged) could have a diminished public health impact and possibly even exacerbate health disparities (Strayhorn et al., 2024). Understanding when and why an EBPI may benefit some youth and not others is critical for identifying and responding to inequities; tests for moderation could thus inform some approaches for achieving net population impacts. The field would also benefit from trials that investigate the equitability of an intervention (Strayhorn et al., 2024). However, given the well-documented barriers to research participation for historically marginalized groups (e.g., mistrust, stigma, logistical challenges; Boyd et al., 2023; Murry et al., 2024), serious consideration must be given to if (and how) the research questions, intervention design, data collection, and intervention implementation procedures are promotive of equitable participation (Schindler, 2024).

### Recommendations and Future Research

Recommendations are organized around two key themes: (1) best practices for subgroup analyses in quantitative research and (2) developing and testing equity-promoting EBPIs. For the former, due to the high risk of selective reporting of subgroup findings in primary studies, all planned interaction/moderation and or within-group subgroup analyses should be specified as part of the preregistration process. Additionally, subgroup effects should be considered during all phases of intervention research studies: design (logic model/theory of change, research questions, sample size), implementation (data collection, analyses of subgroup effects), and dissemination (reporting of confirmatory and exploratory findings, external validity; Breck & Wakar, 2021). Compliance with one or more of the following is further recommended for ensuring credible subgroup effects: (1) preregistration of a whole trial (main effects, hypotheses, analyses, etc.) that also includes preregistered designation of one or two theoretically-justified interaction effects as primary and, ideally, stratification by the preregistered subgroups; (2) corrections for multiple comparisons in the absence of appropriate preregistration; (3) consistency of interaction effects across multiple outcomes and moderators; (4) use of statistical techniques and analyses to ensure robust moderation results; and (5) Replication of significant interactions found in other studies (Bloom & Michalopoulos, 2013; Burke et al., 2015; Elliott et al., 2022; Wallach et al., 2017). Murray and Goodman (2024) provide a collection of articles demonstrating that rigorous methods are available for the design and analysis of cluster RCTs to reduce health disparities, including considerations in multi-level models for subgroup analyses and sample size requirements to test subgroup effects.

Two broad recommendations for designing and testing EBPIs for equitability are as follows. *First, the field should invest in culturally grounded interventions.* Despite interest in incorporating culturally specific content in prevention programming, contemporary prevention science has mostly focused on applying cultural adaptations of EBPIs for minoritized youth (Jackson, 2009; Thier et al., 2020). Far less is known about culturally grounded methods that are intended to contribute organically to developing prevention programs within specific populations and communities (Bo et al., 2023; Lauricella et al., 2016), such as Indigenous youth, families, and communities. Rink et al. (2024) provided case studies addressing how Indigenous partners collaborated with researchers in each stage of the design, implementation, and evaluation of interventions that focus on tribal concepts of the collective, not individuals, and prioritize Indigenous and local knowledge systems over western science methods to reduce health disparities impacting their communities. Further, culturally grounded EBPIs have the potential to anchor culturally focused prevention within certain under-represented groups, thereby addressing disparities for those targeted while also providing a conceptually shorter adaptation bridge for programs focused on related populations and regions. Using this logic, culturally grounded prevention programs developed for Native Hawaiian youth, for example, would have more applicability for adaptation to other Indigenous youth (particularly those within the Pacific region) than programs developed for youth on the continental U.S. and could offer an adaptation template for other Pacific Islander youth (Lauricella et al., 2016; Okamoto et al., 2014).

*Second, establishing criteria for assessing the strength of evidence concerning equity and cultural responsiveness is essential*. Evidence standards (Gottfredson et al., 2015; Steeger et al., 2021) and checklists (Grant et al., 2018; Higgins et al., 2011; Schulz et al., 2010) exist for assessing risk of bias and threats to internal validity in RCTs (i.e., the ability to make causal inferences), but they lack information on how to consider cultural context and equity. Given growing concerns related to the applicability of causal inferences, evaluating external validity is critical (Bryan et al., 2021; Buckley et al., 2023). Equity guidelines exist that the prevention field should consider adopting, drawing upon examples from the Cochrane Handbook and CONSORT-Equity 2017 and PRISMA Equity extensions for reporting trials and reviews (Welch et al., 2012, 2017, 2019). Online Resource Table 6 provides examples of equity and cultural responsiveness items we adapted for consideration in reviews of experimental studies of preventive interventions alongside attributes like risk of bias and threats to internal validity.

### Limitations

Several limitations outlined in Buckley et al. (2023) apply to this study, including (1) human judgment factored into decision-making; (2) absence of robust empirical data specifying in which countries outside of the U.S. the EBPIs in our review were conducted; and (3) infrequent reporting of persons of nonbinary gender identity in samples of primary studies. Additional limitations are as follows. *First*, we did not assess whether the studies in our sample were powered to address subgroup differences, prohibiting us from differentiating between evaluations focused on main effects and those aimed at detecting subgroup differences.

*Second*, our codes do not represent an exhaustive list of historically marginalized populations (for example, individuals with a disability, religious minoritized groups). In addition, coding according to discrete socially constructed groups was necessary given the extent of the information reported in most primary studies — which minimizes the complexity of interactions between multiple forms of disadvantage. While beyond our scope, incorporating variables that disentangle the influence of various social indicators (e.g., SES from race and ethnicity) would facilitate an exploration of intersectionality (i.e., how identities intersect to reflect large social structures of oppression and privilege) and its implications for EBPI findings (Boyd et al., 2023). Meanwhile, inferential models should include variables that represent social mechanisms potentially underlying racial and ethnic differences to unpack how EBPIs might address malleable factors contributing to racial and ethnic disparities (Cheng et al., 2015).

*Third*, we focused on micro-level EBPIs, which reduce challenges for individuals. To advance population-level change, research on structural interventions that describe impacts on equity within the broader social context is needed (Boyd et al., 2023; Murry et al., 2024). For example, macro-level affirmative action policies ensuring fair access to opportunities previously inaccessible to qualified individuals from under-represented groups are one potential avenue. Similarly, meso-level interventions originating from the lived experiences of community members (e.g., community defined evidence) are instrumental for reimagining what constitutes evidence and how this evidence can inform program development and implementation. *Fourth*, our search only included reports screened into Blueprints which limits the generalizability of our findings. This is important considering research indicating minimal overlap between U.S. clearinghouses in EBIs reviewed (Zheng et al., 2022). *Fifth*, a high-quality meta-analysis (Pigott & Polanin, 2019) is needed to make causal inferences, which for adequate statistical power would require expanding beyond the Blueprints database to identify reports. A meta-analysis would overcome another limitation of this research, namely its descriptive nature.

## Conclusion

We found that few experimental reports provided adequate information regarding the potential for EBPIs to promote equity. Issues of equity are always relevant in prevention research because examining effectiveness without investigating the potential to mitigate health disparities can disguise inequities (Strayhorn et al., 2024). Researchers may inadvertently perpetuate the assumption that findings apply to all when they do not explicitly test this assumption (Gaias et al., 2020; Murry et al., 2024). We aim to move the field away from simply focusing on whether an intervention “works,” a false dichotomy that hinders funding decisions. The answer is quite nuanced and depends on several factors (access, content, implementation, etc.). The way forward necessitates examination of not just “what works,” but also “for whom” and in “which settings.”

## Supplementary Information

Below is the link to the electronic supplementary material.

Supplementary file1 (DOCX 60 KB)

## Data Availability

Our dataset is posted on the Open Science Framework (osf.io/huqyf).

## Footnotes

1 Prior to 2010, Blueprints maintained a narrower focus on delinquency and drug use outcomes. To examine a more diverse set of evaluations and emphasize relatively recent research that incorporated up-to-date initiatives of Blueprints, this review excluded evaluations published before 2010. These exclusion criteria are consistent with other studies recently published using the Blueprints database (Buckley et al., 2023; Steeger et al., 2021).

## Materials Availability

Our code book is posted on the Open Science Framework (osf.io/huqyf).

## Code Availability

Our code is posted on the Open Science Framework (osf.io/huqyf).
