# Supplementary material for: Applying an Equity Lens to Evidence-Based Preventive Interventions: a Systematic Review of Subgroup Findings from Experimental Evaluations
Source: Prev Sci. 2025 Jan 17;26(1):93–106. doi: 10.1007/s11121-025-01765-3 (PMC11811249; doi:10.1007/s11121-025-01765-3)
Supplement: Supplementary file 1 — Supplementary file1 (DOCX 60 KB) [file 11121_2025_1765_MOESM1_ESM.docx]

***Prevention Science*** **Online Supplement**

Applying an Equity Lens to Evidence-Based Preventive Interventions: A Systematic Review of Subgroup Findings from Experimental Evaluations

Pamela R. Buckley^†^, PhD, [pamela.buckley@colorado.edu](mailto:pamela.buckley@colorado.edu)

Charleen J. Gust^†^, PhD, [charleen.gust@colorado.edu](mailto:charleen.gust@colorado.edu)

Sarah Gonzalez Coffin, [s.gonzalezcoffin@colorado.edu](mailto:s.gonzalezcoffin@colorado.edu)

Sheba M. Aikawa, [sheba.aikawa@colorado.edu](mailto:sheba.aikawa@colorado.edu)

Christine M. Steeger, PhD, [christine.steeger@colorado.edu](mailto:christine.steeger@colorado.edu)

Fred C. Pampel, PhD, [fred.pampel@colorado.edu](mailto:fred.pampel@colorado.edu)

^†^ *These authors contributed equally to this work.*

**Acknowledgements:** We thank Amanda Ladika for coding.

**Materials availability:** Our code book is posted on the Open Science Framework ([osf.io/huqyf](https://osf.io/huqyf)).

**Code availability:** Our code is posted on the Open Science Framework (osf.io/huqyf).

**Data availability:** Our dataset is posted on the Open Science Framework (osf.io/huqyf).

**Open research statement:** We preregistered with the Open Science Framework (osf.io/huqyf).

**Corresponding author:** Pamela Buckley, Institute of Behavioral Science, University of Colorado Boulder, 483 UCB, Boulder, CO 80309 (e-mail: [pamela.buckley@colorado.edu](mailto:pamela.buckley@colorado.edu)).

**Online Resource Panel 1**

***Blueprints Inclusion Criteria:*** The Blueprints database includes evaluations of interventions for youth designed to: (1) prevent or reduce negative behavioral health outcomes (e.g., mental health problems, substance use, delinquency/crime, and other health-related behaviors); or (2) promote positive development (e.g., academic attainment and prosocial behavioral outcomes). The focus on youth limits interventions to those targeting ages 0-25 years, which include post-secondary education and early employment experiences. Given that the aim of Blueprints is on prevention (including universal, selective, and indicated preventive interventions), the database does not include interventions with a sole focus on evaluating treatment for clinical-level mental health problems, including medical or pharmacological interventions. For each intervention, the database includes experimental evaluations (i.e., randomized control trials, cluster randomized control trials, and quasi-experimental designs) that examine impact (not process evaluations or cost-effectiveness estimates that do not examine condition differences in behavioral outcomes).

**Online Resource Panel 2**

***Search strategy***: Blueprints uses Boolean operators to create multiple search terms as follows. First, several clauses are used to select journals. Second, search terms are applied to locate outcomes for youth relating to physical and mental health, delinquency, educational attainment, prosocial behavior, and problem behavior. Third, these Boolean operators are entered into the Web of Science search engine, which provides subscription-based access to multiple databases with comprehensive citation data for many different disciplines. To locate additional studies, including new evaluations of previously reviewed interventions and new interventions yet to be reviewed, the Blueprints team searches blogs, web pages, other registries, and research organization sites, and accepts nominations from developers and researchers.

**Online Resource Panel 3**

***Methods for Search Terms***

| Search term topic | Search Terms/Clauses |
| --- | --- |
| Journals | JOURNAL OF RESEARCH ON EDUCATIONAL EFFECTIVENESS or Adolescence or Aggression and violent behavior or Aggressive behavior or American journal of community psychology or American journal of public health or American journal of sociology or child abuse & neglect or child development or Crime & delinquency or Criminology or Development and psychopathology or Developmental psychology or Deviant behavior or DRUGS-EDUCATION PREVENTION AND POLICY or Evaluation review or Future of children or Journal of adolescence or Journal of adolescent health or Journal of adolescent research or Journal of applied developmental psychology or Journal of child & adolescent substance abuse or Journal of child psychology and psychiatry and allied disciplines Educational Evaluation and Policy Analysis or American Educational Research Journal or Review of Educational Research or Journal of community psychology or Journal of consulting and clinical psychology or Journal of criminal justice or JOURNAL OF EDUCATIONAL AND PSYCHOLOGICAL CONSULTATION or Journal of family therapy or Journal of family violence or Journal of interpersonal violence or Journal of marriage and the family or JOURNAL OF RESEARCH IN CRIME AND DELINQUENCY or journal of research on adolescence or journal of school psychology or journal of studies on alcohol or JOURNAL OF THE AMERICAN MEDICAL ASSOCIATION or MERRILL-PALMER QUARTERLY-JOURNAL OF DEVELOPMENTAL PSYCHOLOGY or Preventive medicine or Psychological bulletin or School psychology review or Youth & society |
| Mental health | *(program or evaluation or prevention or intervention) and (depression or anxiety or suicide or PTSD or POSTTRAUMATIC-STRESS-DISORDER or obesity or “physical health”) not adult* |
| Delinquency | *(program or evaluation or prevention or intervention) and (violence or aggression or “conduct problem*” or “substance use” or delinquency or “teen* pregnancy” or sex* or bullying or “child maltreatment” or gang or truancy or victimization or school) not adult* |
| Education | *(program or evaluation or prevention or intervention) and (academic or employment or cognitive develop* or dropout or post-secondary or vocational) and (school or community or criminal justice)* |

**Online Resource Panel 4**

***Sample Characteristics (Race, Ethnicity, Gender, and Economic Disadvantage):*** For the sample characteristics, we drafted two sets of codes for each variable. The first set were dichotomous (yes/no) codes used to indicate whether a given study reported individual-level racial, ethnicity, gender, and economic disadvantage data for the sample. The second set of codes, described in more detail below, were used to assess sample composition.

For the initial racial and ethnic composition codes, we followed the most U.S. Census Bureau coding operations at the time of this publication, which treat race and ethnicity as separate identities. Concerning the reporting of race, Census guidelines require the following five categories at minimum: (1) Asian or Asian American; (2) Black or African American; (3) Native American or American Indian or Alaska Native; (4) Native Hawaiian or Pacific Islander; and (5) White (Humes et al., 2011). We added “percent not specified” to our list of racial codes to account for studies that combined two or more racial categories (e.g., Asian and Pacific Islander, a residual other category) or used more detailed classifications not captured by the five Census codes. It should also be noted that the proportion of the sample identifying as Hispanic or Latino was coded as “not specified” for race, as that value was entered into the subsequent ethnicity field. “Percent biracial/multi-racial” was added after our pilot test to account for individuals identifying with multiple races (but not ethnicities).

For ethnicity, two categories (i.e., “Hispanic or Latino” or “not Hispanic or Latino”) was the minimum requirement according to current Census policies (Humes et al., 2011). The codes for gender were: (1) male; (2) female; and (3) persons of nonbinary gender. When coding for race, ethnicity, and gender composition, we entered the proportion of the sample identifying with each of the possible categories.

Finally, to code for economic disadvantage, we opted against relying on income means, medians, or distributions. Instead, to ease interpretation, we considered four proxies: (1) qualifies for the free/reduced-price lunch program; (2) receives Medicaid; (3) Pell-eligible; and (4) qualifies for the Children’s Health Insurance Program. We also considered the proportion of the sample that fell within the federal government’s poverty level, and the lowest income category of an income distribution. We then coded the proportion of the sample fitting with any of these proxies used to indicate economic disadvantage. However, the sample proportions classified as low socioeconomic status (SES) were coded as “not reported” for economic disadvantage, since SES is typically measured as a combination of education, occupation, and income (Mueller & Parcel, 1981).

***Setting Characteristics (Location):*** For the setting characteristics, we were especially interested in the locale where the study was conducted. We focused on urbanicity with rural, suburban, and urban as initial options. Coders were instructed to choose a setting based on the author’s description, and coders could choose more than one type of locale if the study was conducted in multiple settings.

**References:**

Humes, K., Jones, N. A., Ramirez, R. R. (2011). *Overview of race and Hispanic origin: 2010*.

U.S. Department of Commerce, Economics and Statistics Administration. U. S. Census Bureau. Retrieved from: https://www.census.gov/content/dam/ Census/library/publications/2011/dec/c2010br-02.pdf

Mueller, C. W., & Parcel, T. L. (1981). Measures of socioeconomic status: Alternatives and

recommendations. *Child development*, 13-30. <https://doi.org/10.2307/1129211>

**Online Resource Table 1.** Ns and Proportions for Characteristics of Sample EBPI Evaluation Reports

|  | Sample | | |
| --- | --- | --- | --- |
|  | Full:  U.S. and non-U.S. reports | Analysis:  U.S. reports | Sub-Analysis: U.S. reports that conducted subgroup tests^a^ |
|  | N = 292 | N = 240 | N = 100 |
| Design     Randomized control trial (RCT)  Cluster randomized control trial (c-RCT)     Quasi-experimental design (QED)  Article published in an academic journal  Primary age group targeted^b^     Infant (ages 0-2 years)     Preschool (ages 3-4 years)     Elementary school (ages 5-11 years)     Middle school (ages 12-14 years)     High school (ages 15-18 years)     Young adult (ages 19-24 years)     Adult  Setting     Community     Correctional facility     Home     Hospital, medical center     Online     School     Social services  Primary outcome targeted^b^     Adult crime (recidivism)     Educational attainment     Emotional well-being, mental health     Physical health     Positive relationships     Problem behavior | 205 (.70)  78 (.27)  9 (.03)  224 (.77)    12 (.04)  35 (.12)  98 (.34)  114 (.39)  138 (.47)  73 (.25)  15 (.05)    40 (.14)  12 (.04)  27 (.09)  19 (.07)  12 (.04)  174 (.60)  8 (.03)    12 (.04)  105 (.36)  43 (.15)   7 (.02)  7 (.02)  156 (.53) | 175 (.73)  56 (.23)  9 (.04)  176 (.73)    12 (.05)  20 (.08)  72 (.30)  88 (.37)  124 (.52)  65 (.27)  13 (.05)    33 (.14)  10 (.04)  17 (.07)  19 (.08)  11 (.05)  142 (.59)  8 (.03)    10 (.04)  98 (.41)  38 (.16)  7 (.03)  6 (.02)  114 (.48) | 68 (.68)  26 (.26)  6 (.06)  59 (.59)    6 (.06)  7 (.07)  26 (.26)  33 (.33)  57 (.57)  33 (.33)  1 (.01)    14 (.14)  0 (.00)  4 (.04)  7 (.07)  4 (.04)  68 (.68)  3 (.03)    0 (.00)  59 (.59)  9 (.09)  3 (.03)  4 (.04)  34 (.34) |

Note: EBPI – Evidence-Based Preventive Intervention

^a^ Tested for one or more of the following subgroups: race, ethnicity, gender, sexual identity, economic disadvantage, location (rural, urban, suburban), nativity status (foreign-born – yes/no).

^b^ Proportions add to more than 1.0, as EBPIs may target multiple groups and multiple outcomes.

**Online Resource Table 2.** Ns, Proportions, and Descriptive Statistics for Characteristics of Sample EBPI Evaluation Reports

|  | Sample | | |
| --- | --- | --- | --- |
|  | Full:  U.S. and non-U.S. reports | Analysis:  U.S. reports | Sub-Analysis: U.S. reports with subgroup tests^a^ |
|  | N = 292 | N = 240 | N = 100 |
| Reported sample distribution of: | n (Prop) | n (Prop) | n (Prop) |
| Race     Ethnicity     Gender     Economic disadvantage | 227 (.78)  178 (.61)  265 (.91)  100 (.34) | 210 (.88)  178 (.74)  217 (.90)  88 (.37) | 90 (.90)  82 (.82)  91 (.91)  44 (.44) |
| Sample statistics for reported Race: | n = 227  Mean (SD) | n = 210  Mean (SD) | n = 90  Mean (SD) |
| Asian or Asian American        Black or African American        Native American or American Indian  or Alaska Native        Native Hawaiian or Pacific Islander        White        Multi-racial/Biracial        Not specified | .03 (.08)  .30 (.30)  .01 (.02)  .00 (.00)  .38 (.31)  .02 (.04)  .26 (.22) | .03 (.06)  .31 (.30)  .01 (.02)  .00 (.00)  .36 (.31)  .02 (.04)  .27 (.22) | .02 (.06)  .30 (.25)  .01 (.02)  .00 (.00)  .35 (.30)  .01 (.04)  .31 (.21) |
| Sample statistics for reported Ethnicity | n = 178  Mean (SD) | n = 178  Mean (SD) | n = 82  Mean (SD) |
| Hispanic or Latino | .24 (.20) | .24 (.20) | .24 (.18) |
| Sample statistics for reported Gender | n = 265  Mean (SD) | n = 217  Mean (SD) | n = 91  Mean (SD) |
| Female^b^ | .56 (.23) | .57 (.23) | .53 (.13) |
| Sample statistics for reported Economic Disadvantage | n = 100  Mean (SD) | n = 88  Mean (SD) | n = 44  Mean (SD) |
| From a Low-Income Background | .68 (.23) | .70 (.23) | .66 (.23) |

Note: ^a^ Tested for one or more of the following subgroups: race, ethnicity, gender, sexual identity, economic disadvantage, location (rural, urban, suburban), nativity status (foreign-born – yes/no).

^b^ No studies reported another category for persons of nonbinary gender.

**Online Resource Table 3.** Proportions Testing for Subgroup Effects by Culturally Tailored Program (Yes or No) by Subgroup (see research questions 2 and 3)

| Subgroup | Culturally Tailored Program  Analysis Sample (n = 240) | |
| --- | --- | --- |
|  | No | Yes |
| Race  No  Homogeneous Sample  Yes  Ethnicity  No  Homogeneous Sample  Yes  Gender (dichotomous: male/female)  No  Homogeneous Sample  Yes  Sexual Identity  No  Homogeneous Sample  Yes  Economic Disadvantage  No  Homogeneous Sample  Yes  Location (rural, suburban, urban)  No  Homogeneous Sample  Yes  Nativity (foreign-born – yes/no)  No  Homogeneous Sample  Yes | n = 231  .701*  .078  .221  n = 238  .798*  .025  .176  n = 204  .618*  .010  .373  n = 240  .992  .000  .008  n = 215  .740*  .135  .126  n = 226  .982  .000  .018  n = 240  .996  .000  .004 | n = 9  .111  .889  .000  n = 2  .500  .500  .000  n = 36  .056  .944  .000  n = 0  .000  .000  .000  n = 25  .560  .400  .040  n = 14  1.00  .000  .000  n = 0  .000  .000  .000 |

Notes: * Chi-square *p* < .05

Note: We created a dichotomous variable for reports evaluating culturally tailored or non-culturally tailored programs. Then we collapsed the categories to three: examined subgroup differences, used a homogenous sample, or did not examine subgroup differences. This table lists the proportions that examined subgroup differences within the culturally tailored categories.

**Online Resource Table 4.** Proportions Testing for Subgroup Effects by Period of Time (Early Period and Late Period) by Subgroup

| Subgroup | Sample | | | | | |
| --- | --- | --- | --- | --- | --- | --- |
|  | Full  (N = 292) | | Analysis  (N = 240) | | Sub-Analysis  (N = 100) | |
|  | 2010-16  (n = 179) | 2017-23  (n = 113) | 2010-16  (n = 147) | 2017-23  (n = 93) | 2010-16 (n = 57) | 2017-23  (n = 43) |
| Race  No  Homogeneous Sample  Yes  Ethnicity  No  Homogeneous Sample  Yes  Gender  No  Homogeneous Sample  Yes  Sexual Identity  No  Homogeneous Sample  Yes  Economic Disadvantage  No  Homogeneous Sample  Yes  Location  No  Homogeneous Sample  Yes  Nativity Status  No  Homogeneous Sample  Yes | .743*  .117  .140  .888*  .000  .112  .581  .134  .285  1.00  .000  .000  .732  .151  .117  .978  .000  .022  .994  .000  .006 | .726  .044  .230  .743  .062  .195  .478  .168  .354  .982  .000  .018  .743  .133  .124  1.00  .000  .000  1.00  .000  .000 | .687*  .143  .170  .864*  .000  .136  .565  .143  .293  1.00  .000  .000  .694  .184  .122  .973  .000  .027  .993  .000  .007 | .667  .054  .280  .688  .075  .237  .484  .161  .355  .978  .000  .022  .763  .129  .108  1.00  .000  .000  1.00  .000  .000 | .456*  .105  .439  .649  .000  .351  .246  .000  .754  1.00  .000  .000  .561  .123  .316  .930  .000  .070  .982  .000  .018 | .395  .000  .605  .465  .023  .512  .163  .070  .767  .953  .000  .047  .674  .093  .233  1.00  .000  .000  1.00  .000  .000 |

* Chi-square *p* < .05

Note: We created a dichotomous variable for reports published before 2016 (early period) and after 2017 (late period). Then we collapsed the categories to three: examined subgroup differences, used a homogenous sample, or did not examine subgroup differences. This table lists the proportions that examined subgroup differences within “period of time”.

**Online Resource Table 5.** Proportions Registering Prospectively and Retrospectively with and without Subgroup Registration for Sub-Analysis Sample

| Transparency Code | Sub-Analysis Sample:  U.S. reports with subgroup tests^a^ |
| --- | --- |
|  | (N = 100) |
| Registered Prospectively^b^ with Subgroup^c^  Registered Prospectively^b^ without Subgroup^c^  Registered Retrospectively^d^ with Subgroup^c^  Registered Retrospectively^d^ without Subgroup^c^  Not registered | .010  .010  .060  .150  .770 |

Notes:

^a, c^ Tested for one or more of the following subgroups: race, ethnicity, gender, sexual identity, economic disadvantage, location (rural, urban, suburban), nativity status (foreign-born – yes/no).

^b^ **Registered prospectively** means that study participants were enrolled and the study was documented in a public registry before the outcomes relevant to the study’s objectives were known or analyzed. This practice enhances the reliability and credibility of research findings by ensuring transparency and reducing the potential for bias.

^d^ **Registered retrospectively** means that the study was entered into a public registry after data collection had started or after the study was completed. While retrospective registration provides transparency post-facto, it is generally considered less desirable than prospective registration because it can introduce bias. For example, researchers might selectively report outcomes or change their analysis plan based on the data collected, potentially compromising the study's scientific integrity.

**Online Resource Table 6.** Blueprints for Healthy Youth Development is an online clearinghouse of evidence-based preventive interventions (Mihalic & Elliott, 2015). Blueprints currently assesses intervention specificity, internal validity (Steeger et al., 2021) and dissemination readiness (Buckley et al., 2020) in conducting an evidence review of preventive interventions for youth. These items are listed in sections III, IV, and V below. We propose to add additional items to assess equity and cultural responsiveness of preventive interventions, with sample items listed in sections I and II below.

1. **Proposed sample items** for assessing the strength of evidence concerning **Equity**, adapted from the CONSORT-Equity 2017 extension and elaboration for better reporting of health equity in RCTs (Welch et al., 2017):
2. “Equity” is in the title, program description, theory of change, and/or program logic model.
3. Program exclusively focuses on individuals or a population who experience socially structured disadvantage.
4. Program is culturally tailored (understanding that culture plays a significant role in shaping individuals’ behaviors, attitudes, and perceptions, the program is culturally adapted, deep-structure adapted, or culturally tailored).
5. Sample includes participants who experience social disadvantage within the setting and context of the evaluation.
6. Sample consists of mixed racial and ethnic groups and differential impacts of the intervention are assessed, including whether analyses to estimate heterogeneity among subgroups were preregistered and powered to detect differences.
7. Report details of subgroup hypotheses across PROGRESS-Plus characteristics, an acronym used by the Cochrane Collaboration to identify characteristics that stratify health opportunities and outcomes.
8. **Proposed sample items** for assessing the **Cultural Relevance** of intervention content and delivery throughout the design, evaluation, and implementation phases (AECF, 2014; Andrews et al., 2019; Samuels et al., 2009; Wiggins & Sileo, 2020):
9. The developers and implementers have a deep understanding of the cultural backgrounds of the target population; they are knowledgeable about the cultural norms, traditions, and values of the group they are serving.
10. Communities and individuals from the target cultural group were central to the development and evaluation of the program.
11. Illustrations are presented in a way that participants can see themselves, their surroundings, and their values.
12. Research-informed guidance is provided to assist with appropriate cultural adaptations in aligning the program with the cultural preferences and sensitivities of the target group; this may involve modifying language, images, examples, and activities to be more culturally relevant.
13. The program is delivered in the participants’ first language.
14. The content, illustrations, and language directly communicate respect for the participants’ native and/or home culture.
15. The content, illustrations, and language explicitly communicate hope, care, and positive regard to participants.
16. The program is financially accessible to all youth and families, regardless of their economic status; this includes evaluating program fees, transportation options, and the availability of scholarships or subsidies for participants who come from a low-income background.
17. Program information is easily accessible and available in multiple formats (e.g., print, digital, oral) to accommodate different communication preferences and literacy levels.
18. **Current criteria** for Blueprints **Intervention Specificity** standard of evidence (Mihalic & Elliott, 2015):
    1. The intended participants to receive the intervention are clearly identified.
       1. The relevant sociodemographic characteristics (age, gender, ethnic group, socio‐economic status, urban/suburban/rural residence) of those targeted by the intervention are stated.
       2. If the intended participants are those who have been screened based upon some characteristic(s) (e.g., a risk condition, protective factor status, a minimum level of the study outcome, or some personal or family attribute), these screening criteria and the screening process must be fully described.
       3. All inclusion or exclusion criteria for program participation must be noted.
    2. The intervention’s theoretical rationale or logic model is discussed explaining how the intervention is expected to have a positive effect on intended outcomes and whether/how changes in risk or protection factors will affect the specified outcome(s).
    3. There is documentation of the intended intervention structure, content and delivery process. A clear description of the planned intervention is reported, such as what service, activity or treatment is provided, to whom, by whom, over what period, with what intensity and frequency, and in what setting. This can include the:
       1. Content of the intervention (e.g., information, advice, training, money, advocacy).
       2. Nature of the provider (e.g., social worker, teacher, psychologist, volunteer).
       3. Duration of the intervention (e.g., 3 hours, 6 weeks, a school year).
       4. Length of participation at each session/contact (e.g., 2 hours).
       5. Frequency of sessions/contacts (e.g., daily, weekly, monthly).
       6. Setting (e.g., school, community center, health clinic).
       7. Mode of delivery (e.g., group‐based, one‐to‐one). In the case of a multi‐component intervention – for example, one that has components for children only, parents only, and children and parents together – it is necessary for each component to be described in these terms.
19. **Current Internal Validity** criteria for Blueprints “evaluation quality” standard of evidence (Steeger et al., 2021):
20. Does the study have a high-quality design that is free of threats to the random assignment (e.g., consent after randomization, design confound)?
21. Does the study clearly describe the sample size at each stage of data gathering so that attrition from the randomized sample can be calculated at posttest and each follow-up?
22. Is measurement of the outcomes done independently from the delivery of the intervention, are outside raters blind to condition, and do participant reports avoid social desirability and demand bias?
23. Are the measures reliable and valid as shown by acceptable psychometric properties of the measures (e.g., interrater reliability, Cronbach’s alpha)?
24. Does the study use an intent-to-treat analysis by attempting to follow and analyze all subjects as assigned to their original condition?
25. Was the analysis done at the proper level, with multilevel statistical methods or other adjustments for clustering, when clusters rather than individuals were randomized?
26. Does the analysis control for baseline outcome measures with the use of change scores, baseline outcomes as covariates, or group-by-time interactions?
27. Does the analysis demonstrate baseline equivalence between conditions with statistical tests, effect sizes, or other measures of condition differences across all baseline sociodemographic and outcome measures?
28. Does the study demonstrate with statistical tests that any attrition beyond minimal levels is unrelated to group assignment, sociodemographic characteristics, or baseline outcomes?
29. **Current criteria** for Blueprints **Dissemination Readiness** standard of evidence (Buckley et al., 2020):
30. There is a clear description of the activities of the intervention.
31. The version of the intervention that met standards for evaluation quality is currently available for sites wishing to implement it. At a minimum, there is a contact person with knowledge of the intervention’s theoretical rationale and activities that were evaluated and shown to improve outcomes. Ideally, there is an up-to-date website and program materials can be ordered.
32. There is a curriculum, protocols and/or explicit implementation procedures with instructions that specify the intervention content and guide the implementation of the intervention. Ideally, this includes a manual or series of manuals specifying in detail what the intervention comprises.
33. There is an established system for monitoring implementation fidelity, including fidelity measures. Ideally, this includes measures to assess pre/post changes in outcomes.
34. There are levels of formal training or qualifications for those delivering the intervention. Ideally, this includes training provided by certified instructors. It also ideally includes ongoing technical assistance and coaching.
35. The financial resources required to deliver the intervention are specified. Ideally, there is a description of costs associated with implementing the program, including: start‐up costs; intervention implementation costs; intervention implementation support costs, such as technical assistance and training; and costs associated with fidelity monitoring and evaluation. A breakdown of cost for these separate components, when appropriate, is identified.
36. There is reported information on the human resources required to deliver the intervention. Ideally, there is a description of staff resources needed to deliver the intervention, including required staff ratios, the required level of qualifications and skills for staff, and the amount of time they will need to allocate (to cover delivery, training, supervision, preparation and travel).

**References:**

Andrews, K., Parekh, J., and Peckoo, S. (2019). *How to embed a racial and ethnic equity*

*perspective in research: Practical guidance for the research process.* Child Trends Working Paper. Retrieved from: <https://cms.childtrends.org/wpcontent/uploads/2019/09/RacialEthnicEquityPerspective_ChildTrends_October2019.pdf>

Annie E. Casey Foundation, AECF (2014). *Race equity and inclusion action guide.* Annie E.

Casey Foundation: Baltimore, MD. Retrieved from: <https://www.aecf.org/resources/race-equity-and-inclusion-action-guide>

Buckley, P. R., Fagan, A. A., Pampel, F. C., & Hill, K. G. (2020). Making evidence-based

interventions relevant for users: A comparison of requirements for dissemination readiness across program registries. *Eval Rev, 44*(1), 51-83.

Mihalic, S. F., & Elliott, D. S. (2015, Feb). Evidence-based programs registry: Blueprints for

Healthy Youth Development. *Eval Program Plann, 48*, 124-131.

Samuels, J., Schudrich, W., & Altschul, D. (2009). *Toolkit for modifying evidence-based*

*practice to increase cultural competence.* Research Foundation for Mental Health: Orangeburg, NY. Retrieved from: <https://calmhsa.org/wp-content/uploads/2013/10/ToolkitEBP.pdf>

Steeger, C. M., Buckley, P. R., Pampel, F. C., Gust, C. J., & Hill, K. G. (2021). Common

methodological problems in randomized controlled trials of preventive interventions. *Prev Sci, 22*(8), 1159-1172.

Welch, V. A., Norheim, O. F., Jull, J., Cookson, R., Sommerfelt, H., Tugwell, P., Equity, C., &

Boston Equity, S. (2017). CONSORT-Equity 2017 extension and elaboration for better reporting of health equity in randomised trials. *BMJ, 359*, j5085.

Wiggins, M.E. & Sileo, A. (2020). *What's the role of equity in evaluation policy?* The Forum for

Youth Investment. Retrieved from: <https://forumfyi.org/wp-content/uploads/2020/02/Equity-in-Evaluation-Policy-Winter-2020.pdf>
